# Mitochondrial *AtTrxo1* is transcriptionally regulated by AtbZIP9 and AtAZF2 and affects seed germination under saline conditions

**DOI:** 10.1093/jxb/erx012

**Published:** 2017-02-10

**Authors:** Ana Ortiz-Espín, Raquel Iglesias-Fernández, Aingeru Calderón, Pilar Carbonero, Francisca Sevilla, Ana Jiménez

**Affiliations:** 1 Departamento de Biología del Estrés y Patología Vegetal, CEBAS-CSIC, Campus Universitario de Espinardo, 30100-Murcia, Spain; 2 Centro de Biotecnología y Genómica de Plantas (CBGP; UPM-INIA), Campus de Montegancedo, Universidad Politécnica de Madrid, Pozuelo de Alarcón, 28223-Madrid, Spain

**Keywords:** *Arabidopsis thaliana*, *AtTrxo* gene family, *AtTrxo1* gene expression, germination, ROS homeostasis, saline conditions, transcriptional regulation

## Abstract

Mitochondrial thioredoxin-*o* (AtTrx*o*1) was characterized and its expression examined in different organs of *Arabidopsis thaliana*. *AtTrxo1* transcript levels were particularly high in dry seeds and cotyledons where they reached a maximum 36 h after imbibition with water, coinciding with 50% germination. Expression was lower in seeds germinating in 100 mM NaCl. To gain insight into the transcriptional regulation of the *AtTrxo1* gene, a phylogenomic analysis was coupled with the screening of an arrayed library of Arabidopsis transcription factors in yeast. The basic leucine zipper AtbZIP9 and the zinc finger protein AZF2 were identified as putative transcriptional regulators. Transcript regulation of AtbZIP9 and AtAFZ2 during germination was compatible with the proposed role in transcriptional regulation of *AtTrxo1*. Transient over-expression of AtbZIP9 and AtAZF2 in *Nicotiana benthamiana* leaves demonstrated an activation effect of AtbZIP9 and a repressor effect of AtAZF2 on *AtTrxo1* promoter-driven reporter expression. Although moderate concentrations of salt delayed germination in Arabidopsis wild-type seeds, those of two different *AtTrxo1* knock-out mutants germinated faster and accumulated higher H_2_O_2_ levels than the wild-type. All these data indicate that AtTrxo1 has a role in redox homeostasis during seed germination under salt conditions.

## Highlight

Mitochondrial AtTrxo1 has a role in redox homeostasis during seed germination under salt conditions, and it could act as a possible sensor of saline stress and an inducer of H_2_O_2_ accumulation, independently of other ROS parameters

## Introduction

Plant cells generate reactive oxygen and nitrogen species (ROS and RNS, respectively) during plant development, including maturation and germination of seeds, and they can act as signaling molecules (El-Maarouf-Bouteau and Bailly, 2008; Sanz *et al.*, 2015). The germination of seeds proceeds in two different steps: (i) germination *sensu stricto*, spanning from the start of water uptake to radicle emergence; and (ii) reserve mobilization, which is considered a post-germination process (Bewley, 1997; Nonogaki, 2014). In *Arabidopsis thaliana*, germination *sensu stricto* involves rupturing of the testa and breakage of the micropylar endosperm, which occurs mainly due to the weakening of the endosperm cell walls by mannanases and other hydrolytic enzymes (Iglesias-Fernández *et al.*, 2011, 2013; González-Calle *et al.*, 2015). The emergence of the radicle marks the onset of post-germination events, and growth is supported by the hydrolysis of reserve compounds (proteins, lipids, carbohydrates) until the seedling becomes fully photosynthetic (Vicente-Carbajosa and Carbonero, 2005; González-Calle *et al.*, 2014; Iglesias-Fernández *et al.*, 2014).

To avoid the harmful effects of ROS/RNS, plants have evolved a range of redundant and elaborate mechanisms that involve metabolites and enzymes under oxidative and nitrosative stress responding to developmental and environmental cues (Mittler *et al.*, 2011; Noctor and Mhamdi, 2014; Sevilla *et al.*, 2015). Thioredoxins (Trxs) are ubiquitous small proteins (around 12 kDa) with oxidoreductase activity, containing two cysteines in the redox-active center that regulate the function of target proteins. Trxs are a key factor in maintaining protein dithiol/disulphide homeostasis, which modulates redox signaling during development and stress adaptation (Meyer *et al.*, 2012; Lázaro *et al.*, 2013). In plants, there are at least 10 families of Trxs, with more than 40 members (Meyer *et al.*, 2012; Traverso *et al.*, 2013), and the presence of at least 29 *Trx* genes has been reported in the Arabidopsis genome (Meyer *et al.*, 2009; Belin *et al.*, 2015). Trxs are present in almost all cellular compartments, including chloroplasts, mitochondria, apoplast, cytosol and nuclei (Meyer *et al.*, 2012; Traverso *et al.*, 2013). To date, the best-known mitochondrial Trxs are of the *o*-type, but the pea Trxo1 has been also found in the nucleus (Martí *et al.*, 2009). Pea mitochondrial PsTrx*o*1 has been reported to be involved in the response mechanism against salt stress in pea leaves, in addition to mitochondrial PrxIIF and other antioxidant enzymes (Martí *et al.*, 2011). Mitochondrial Trx*o*1 may function in the reductive activation of citrate synthase, alternative oxidase (AOX), and PrxIIF (Umbach and Siedow, 1993; Martí *et al.*, 2011) thereby favouring flux through the Krebs cycle and the respiratory chain over fermentative processes (Rhoads *et al.*, 1998; Florez-Sarasa *et al.*, 2014). Interestingly, a recent study by Daloso *et al.* (2015) has revealed that mitochondrial enzymes of the TCA cycle are redox regulated, since in the mutant *AtTrxo1* the enzymatic activities of ATP-cytrate lyase (ACL) and succinyl-CoA ligase (SCoAL) are drastically decreased, suggesting that Trxo1 promotes differential redox regulation of these enzymes.

In plant mitochondria, the presence of thioredoxin-dependent peroxirredoxins (Prxs) and sulfiredoxins (Srxs) has also been described (Dietz *et al.*, 2006; Barranco-Medina *et al.*, 2007, 2008). In this organelle, Trxs coupled with Prxs scavenge H_2_O_2_ (Konig *et al.*, 2002; Barranco-Medina *et al.*, 2007), and Srx (which is a small thiol reductase) catalyzes the retro-reduction of hyperoxidized (sulfinic) Prx in an ATP-dependent manner, similar to that proposed for other Srxs (Rey *et al.*, 2007; Iglesias-Baena *et al.*, 2010, 2011). In the past decade, our understanding of the chloroplast and cytosol Trx systems has grown significantly. Trx*h* isoforms constitute the largest group in the Trx family in Arabidopsis and extensive work on its function has implicated it in the seed germination process (Montrichard *et al.*, 2003; Serrato and Cejudo, 2003; Pulido *et al.*, 2009). In contrast, relatively few studies have addressed the possible involvement of the plant Trx*o* system in germination and no data are available about its transcriptional regulation. Transcriptional regulation of gene expression is driven by short DNA sequences (*cis*-elements) in gene promoters and by transcription factors (TFs), proteins that interact with them. Comparison of the promoter sequences of orthologous genes facilitates the finding of these *cis*-elements, which are conserved through evolution (‘phylogenomics’), and these are used as baits for the screening of an arrayed library of Arabidopsis TFs (Y1H, yeast one-hybrid assays; Castrillo *et al.*, 2011).

In this study, the molecular characterization of the gene *AtTrxo1* from Arabidopsis has been carried out, with special emphasis on its transcriptional regulation and in its role in seed germination. For that purpose, a bioinformatic search for its putative orthologous genes and corresponding promoters within the Brassicaceae family has been done and a conserved promoter *cis*-element has been used as the bait to look for interacting TFs in a yeast library of circa 1200 ORF TFs from Arabidopsis (Castrillo *et al.*, 2011). Among the different interacting TF proteins, a basic-leucine zipper AtbZIP9 and a zinc finger protein AZF2 have been identified as possible transcriptional regulators of the *AtTrxo1* gene. To explore the potential physiological role of this Trx*o*1, we have carried out a comparison of the wild-type and two *AtTrxo1* knock-out mutants, and also examined the effect of lacking Trx*o*1 on plant development under saline (100 mM NaCl) conditions, including specifically a deeper study of the germination process. A comparison of the germination kinetics of the wild-type and the knock-out mutants in 100 mM NaCl demonstrated that the mutants germinate faster than the wild-type seeds under these stress conditions.

## Materials and methods

### Plant material, growth conditions, and germination assays

Seeds of *Arabidopsis thaliana* ecotype Columbia (Col-0; the wild-type, WT) and two different T-DNA insertion mutants [knock-out (KO) *AtTrxo1*: SALK_143294C and SALK_042792] were obtained from the European Arabidopsis Stock Centre (NASC, http://Arabidopsis.info/). The homozygous plants for these T-DNA insertions were selected by PCR using gene-specific primers and a primer derived from the left border (LBb1.3) of the T-DNA (http://signal.salk.edu/tdnaprimers.2.html; see Supplementary Table S1 available at *JXB* online).

Plants (one per pot) were grown in substrate containing perlite:peat soil (1:3, v:v) under controlled conditions of light (150 µmol m^−2^ s^−1^ PAR), photopheriod (16/8 h light/dark), relative humidity (60%), and temperature (23/18 °C light/dark). Study of the response of the WT and KO mutant plants to salt stress was carried on 7-d-old plants exposed to water (control) and 100 mM NaCl twice a week during plant development. Plants were harvested after 28 and 42 d of growth.

Arabidopsis seeds were surface-sterilized and germinated in 0.5× Murashige and Skoog solidified medium including vitamins (Duchefa-Biochemie, Haarlem, The Netherlands) essentially as described in Iglesias-Fernández *et al.* (2014). Seeds (WT and KO mutants) were after-ripened at 21 °C and 30% relative humidity for 1 month before germination assays were performed.

Three replicates of 100 after-ripened seeds were imbibed in 90-mm Petri dishes on Whatman No.1 filter paper moistened with 3 ml of 100 mM NaCl (controls in 3 ml H_2_O). This NaCl concentration was chosen from a range of 10, 50, 75, 100, and 150 mM because it allowed the complete germination of seeds of WT and KO mutants while presenting different germination kinetics (Supplementary Fig. S2). Germination was under long-day conditions (16/8 h light/dark; light intensity of 150 µmol m^−2^ s^−1^ PAR). Seeds were not surface-sterilized in order to avoid influencing their dormancy status, and were considered germinated when radicle protrusion was visible under a magnifying lens. Germination tests were performed four times.

### Generation of transgenic lines and histochemical β-glucuronidase (GUS) assays

The transgenic reporter lines *PAtTrxo1::uidA* were produced by fusing 1011 bp of the *AtTrxo1* promoter (amplified from Arabidopsis genomic DNA by nested PCR using oligonucleotide pairs as shown in Supplementary Table S1) to the *uidA* reporter gene. The promoter fragment was cloned into the pDONR221 vector by the Gateway^®^ BP recombination and then transferred by Gateway LR^®^ recombination (Invitrogen, http://www.invitrogen.com) into the destination vector pMDC163. This construct was introduced into *Agrobacterium tumefaciens* strain C58C1 GV3101 by electroporation and then used to transform Arabidopsis (Col-0) by the floral dip method (Clough and Bent, 1998).

Qualitative GUS staining assays were performed as described by Jefferson *et al.* (1987) and Stangeland and Salehian (2002), and were visualized under a magnifying lens (Leica, Wetzlar, Germany).

### Bioinformatic tools

The sequences from five different Brassicaceae (*A. thaliana*, *A. lyrata*, *Brassica rapa*, *Capsella rubella*, and *Eutrema salsugineum*) and two Leguminosae (*Phaseolus vulgaris* and *Glycine max*) species were obtained from the Phytozome v8.0 Database (http://www.phytozome.net;Goodstein *et al.*, 2012). The sequence of the pea *PsTrxo1* has been described in Martí *et al.* (2009). The deduced amino acid sequences of the 13 *Trxo* genes were used to construct a phylogenetic dendrogram. The alignment of these sequences was carried out with the CLUSTALW programme (Thompson *et al.*, 1994) prior to the phylogenetic analysis, which was done by the neighbour-joining method with the MEGA 4.0 software (Tamura *et al.*, 2007), using a bootstrap analysis with 1000 replicates, complete deletion, and the Jones–Taylor–Thornton matrix as settings. The conserved motifs within the deduced protein sequences of the 13 Trx*o* were identified with the MEME program (http://meme-suite.org/tools/meme;Bailey *et al.*, 2009). Mitochondrial signal peptides and their cleavage sites were predicted using MitoProt (ExPASy tools, http://www.expasy.org/tools). The promoter sequences of *Trxo*1 from *A. thaliana*, *A. lyrata*, and *C. Rubella* were used to create pair-wise alignments (phylogenomics) using the software mVISTA Shuffle-LAGAN (http://genome.lbl.gov/vista/mvista/submit.shtml;Frazer *et al.*, 2004) and T-Coffee (http://www.ebi.ac.uk/Tools/msa/tcoffee;Notredame *et al.*, 2000).

### Yeast one-hybrid (Y1H) assays

Yeast one-hybrid screenings were performed essentially as described by Castrillo *et al.* (2011). The *AtTrxo1-B2*-element was amplified by PCR using specific primers that contained *Xma*I and *Xba*I restriction sites (Supplementary Table S1), and this PCR product was cloned into the pTUY1H plasmid upstream of a *HIS3* reporter gene to be used to transform *Saccharomyces cerevisiae* Y187α (MAT-α) cells. Positive colonies were visible after 2–5 d of incubation at 28 °C in a selection medium lacking leucine (L), tryptophan (W), and histidine (H) under increasing concentrations of the inhibitor 3-AT (3-amino-1,2,4- triazole; Sigma, St Louis, MO, USA).

### Transient trans-activation assays

A set of three different constructs, derived from the promoter of *AtTrxo1*, were fused to the reporter *uidA* gene (GUS) and used to transform *A. tumefaciens* (strain C58C1 GV3101) for transient expression assays with or without the effector construct *P35S::AtbZIP9, P35S::AtAZF2* and these were used to infiltrate *Nicotiana benthamiana* leaves. In addition, the leaves were also infiltrated with *P35S::LUC* (luciferase) for normalization and with *P35S::P19* to avoid silencing (Jefferson *et al.*, 1987; Voinnet *et al.*, 2003). Relative GUS/LUC activities were determined by fluorescence and luminescence using a Genios Pro 96/384 multifunction microreader (TECAN^®^; Tecan Group, Männedorf, Switzerland). Three independent transformation experiments were done for each construct.

### Real-time quantitative PCR assays

Total RNA was purified from different organs of Arabidopsis, including seeds at several time-points during germination (Oñate-Sánchez and Vicente-Carbajosa, 2008), and used to synthesized cDNA from 1-μg RNA samples (RT-PCR Kit from Roche Applied Science, Mannheim, Germany). The specific primers for the RT-qPCR analyses are shown in Supplementary Table S1 and the expression of the *Actin 8* (ACT-8, *At1g49240*) gene was used to normalize the data (Graeber *et al.*, 2011). Eco-Real-Time PCR System (Illumina, San Diego, CA, USA) was used and for each 10-μl reaction: 1 μl cDNA sample was mixed with 5 μl of FastStart Universal SYBR Green Master (Roche Applied Sciences), 0.25 μl of each primer (final concentration 500 nM), plus sterile water up to the final volume. The thermal-cycling conditions were 95 °C for 10 min, 40 cycles for 10 s at 95 °C, and 30 s at 60 °C. The melting curve was designed to increase from 55 to 95 °C and primer efficiencies were estimated from a calibration dilution curve and slope calculation (Supplementary Table S1). This analysis was performed with three different biological samples for each time point. Expression levels were determined as the number of cycles needed for the amplification to reach a threshold fixed in the exponential phase of the PCR reaction (Ct; Pfaffl, 2001).

### Determination of ROS parameters: H_2_O_2,_ lipid peroxidation and protein oxidation

Hydrogen peroxide content was measured in seeds using the eFox method as described by Camejo *et al.* (2011). Samples of 50–100 mg of seeds at different times of germination were homogenized in liquid nitrogen, re-suspended in 1 ml of acid acetone (0.13% sulphuric acid in acetone) and frozen again in liquid nitrogen. After defrosting, the cellular mixture was centrifuged at 10 000 *g* for 10 min at 4 °C, and the supernatant was mixed (1:5, v/v) with the assay solution (250 μM ferrous ammonium sulphate, 25 mM H_2_SO_4_, 100 μM xylenol orange, 100 mM sorbitol). After 45 min incubation at room temperature, the peroxide-mediated oxidation of Fe^2+^ to Fe^3+^ was determined by measuring the absorbance at 560 nm of the Fe^3+^ xylenol orange complex formed.

The level of lipid peroxidation in 50–100 mg samples of seeds was estimated by determining the concentration of ThioBarbituric Acid-Reactive Substances (TBARS) as described by Cakmak and Horst (1991).

Protein oxidation (carbonyl protein content) from 50 mg samples of seeds was measured as described by Vanacker *et al.* (2006) by reaction with 2,4 dinitrophenylhydrazine (Levine *et al.*, 1990). Total soluble proteins were measured by the Bradford method, using Bovine Serum Albumin (BSA) as the standard (Vanacker *et al.*, 2006).

### Statistical analyses

Experiments were conducted in a completely randomized design. The results presented are the mean of at least three biological replicates from each experiment, and all the experiments were repeated at least three times. Data were subjected to ANOVA (one factor) using Tukey’s test (*P*<0.05), using the IBM SPSS Statistics 20 programme.

## Results

### The thioredoxin-*o* (Trx*o*) gene subfamily

**Fig. 1.**
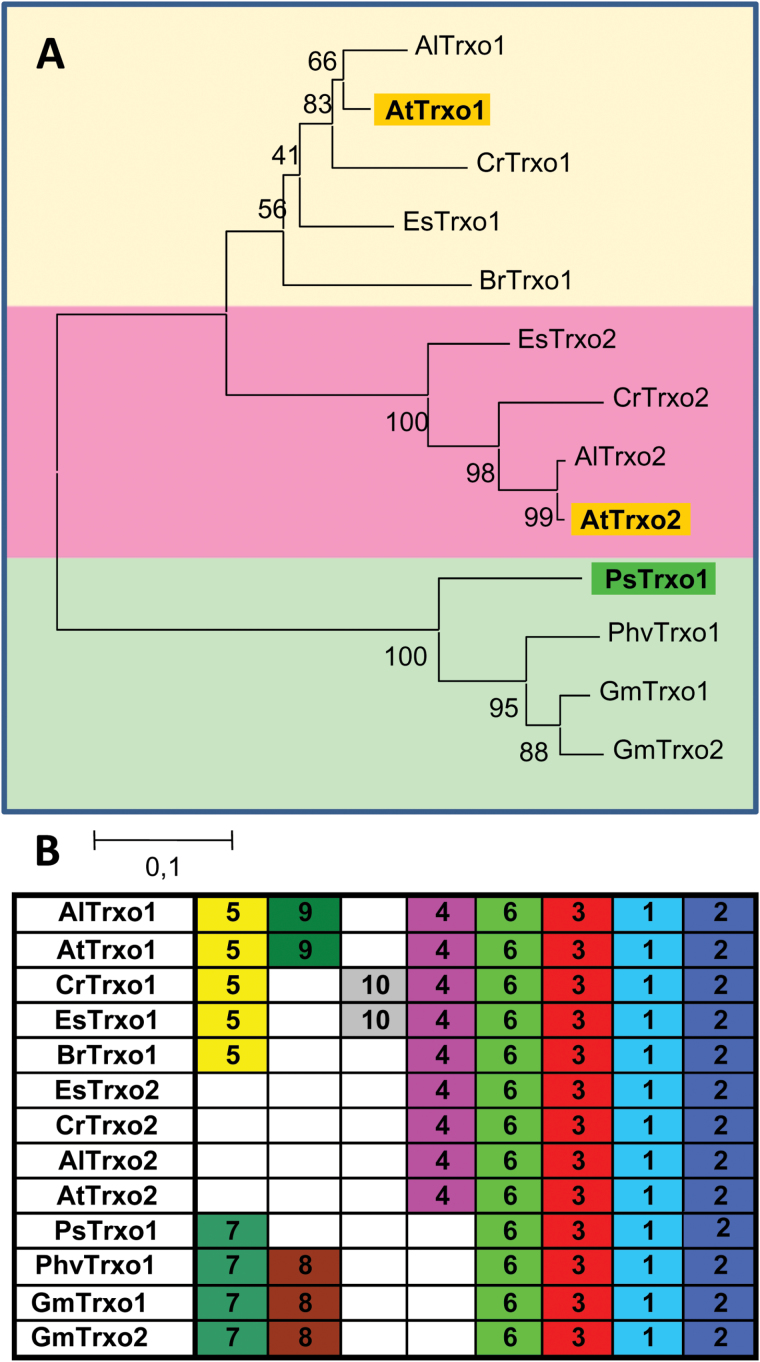
(A) Phylogenetic dendrogram with the deduced protein sequences of AtTrxo1, AtTrxo2, and PsTrxo1 and their orthologs from other Brassicaceae and Leguminosae as determined from databanks (see text for details); bootstrapping values are indicated on the branches. Abbreviations: Al, *Arabidopsis lyrata*; At, *A. thaliana*; Cr, *Capsella rubela*; Br, *Brassica rapa*; Es, *Eutrema salsugineum*; Ps, *Pisum sativum*; Phv, *Phaseolus vulgaris*; Gm, *Glycine max*. Accession numbers: *AlTrxo1* (482466), *AtTrxo1* (AT2G35010), *CrTrxo1* (Carubv 10024096m.g), *EsTrxo1* (Thahalv10017264m), *BrTrxo1* (021942), *EsTrxo2* (Thahalv10008976m), *CrTrxo2* (Carubv 10010516m), *AlTrxo2* (473345), *AtTrxo2* (AT1G31020), *PsTrxo1* (EMBL: Q257C6), *PhvTrxo1* (091018134m), *GmTrxo1* (05g26300.1), *GmTrxo2* (08g09210.1). (B) Schematic distribution of conserved motifs among the deduced protein sequences in the phylogenetic tree in (A), identified by means of the MEME analysis. (This figure is available in colour at *JXB* online.)

Previous studies on the thioredoxins of *Pisum sativum* (Leguminosae) reported for the first time the presence of a gene encoding a mitochondrial and nuclear Trx*o* isoform (Martí *et al.*, 2009). In a further investigation, we searched for its putative Arabidopsis orthologous gene(s) using bioinformatic tools. Two *thioredoxin-o* genes, *AtTrxo1* and *AtTrxo2* (loci *At2g35010* and *At1g31020*, respectively) are present in the Arabidopsis genome (Laloi *et al.*, 2001), and a non-redundant compilation of deduced Trx*o* orthologs in other Brassicaceae (*A. lyrata*, *C. rubella*, *E. salsugineum*, *B. rapa*) and other Leguminosae (*P. vulgaris* and *G. max*) genomes have been annotated and used to construct a phylogenetic un-rooted tree using the neighbour-joining algorithm (Fig. 1A). The pair-wise amino acid similarities (>50%) clearly delimit three clades: two containing the Brassicaceae sequences, and one containg the Leguminosae Trx*o* proteins. The occurrence of common motifs analysed by the MEME software further support the three clades (Fig. 1B and Table 1). Proteins in the first clade, sharing motifs 1, 2, 3, and 6 with the other Trx*o* sequences, also share motifs 5 and 4. Members of the Leguminosae group have motif 7 in common instead of motif 5 and lack motif 4. Moreover, the position of the predicted intron–exon gene structures in all *Trxo* orthologous sequences is conserved among members of the same clade (Supplementary Fig. S1).

**Table 1.** Sequences of conserved amino-acid motifs (MEME analysis; Bailey *et al.*, 2009) of the 13 deduced Trx*o*1 and Trx*o*2 protein sequences shown in Fig. 1. The conserved amino acids of the active site (WCGPC) are in bold. Motifs containing predicted mitochondrial localization signals according to the MitoProt software (Claros and Vicens, 1996) are in italics

**Motif**	**E-value**	**Consensus sequences**
1	7.3e-472	K[AV][QR]D[GD]***S***L[PH][***S***A][VI]FYFTA[AV]**WCGPC**R[FL]I[SA]P[VI][IV][GLV]ELSK[KQ]YPDVTTYK[VI]DID[EQ][GE][GA][LI]
2	6.8e-266	[IL][GS]KL[NQ][IV][ST][AS]VPTL[HQ]FF[KQ][GN]G[SVK]K[KA][AGD]E[ILV]VG[AV]DV[ATV][KR]LK[NS][LIV][MT]E[QK]L[YF]K
3	1.0e-091	AG[GDA][EPR][SN][GDS][VF]V[LIV][VL][KN]SE[EA]EF[NI][NS][AI][LM][ST]
4	4.6e-082	[PQ][NW]S[INM][FSP][SH][QL]I[AG]RNS[LF][FL][AT]AST[IVF][GY][APV]S[IT][ED]FNF[SL]NTS
5	1.0e-040	*MKG[NS][WFL]SI[VI]R[QK][VFI][LF][HQ]R[RQ]FSTLRSS[TRS][PT]*
6	3.4e-026	[LF][PHL]H[RS]RS[LF][CS]
7	6.2e-022	*[MG][AT]R[NL][LW][VIL][VF]RSLALRH[AV][IM]KN[RT][VG][RL][PT][LI][FLS][FT][NH][RAT][HILN][LH]*
8	4.1e+002	[TF]A[TA]A[TI]ASS[QR]LSLLP
9	1.7e+004	RLSTS[IV]RPLV
10	9.9e+004	[AS]RP[LS][AM]L

### AtTrxo1 *expression in vegetative and reproductive organs*

**Fig. 2.**
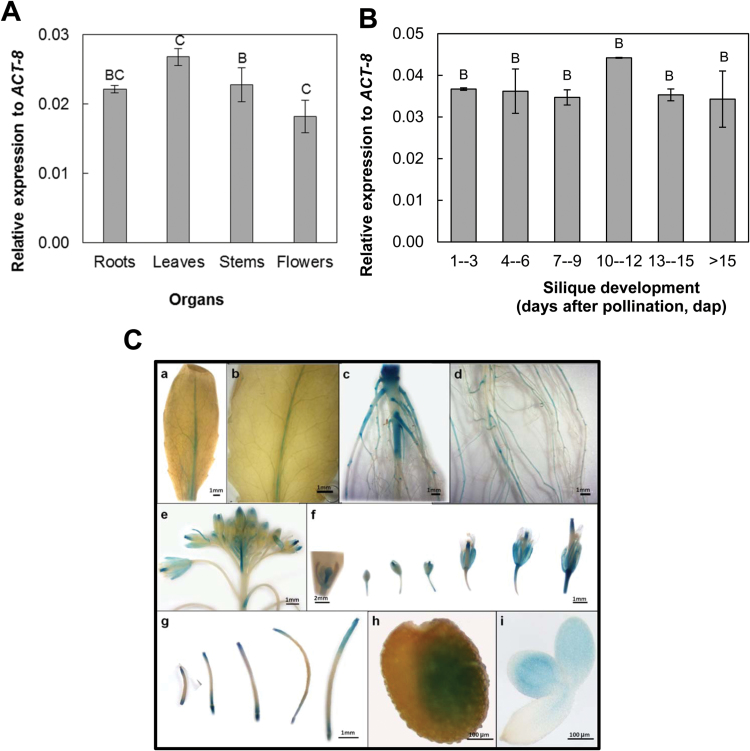
Expression analysis of the *AtTrxo1* gene (relative to *ACT-8*) as determined by RT-qPCR (A) in different organs of the Arabidopsis plant, and (B) during silique development. (C) Expression of β-glucuronidase (GUS) activity driven by the *AtTrxo1* whole promoter (–1047 bp) fused to the reporter *uidA* (GUS) gene in transgenic lines of Arabidopsis: (a, b) rosette leaf; (c, d) root system; (e) inflorescence; (f) floral to fruit development; (g) silique development; (h) seed after 24 h of imbibition; (i) same as (h) without seed coat.

*AtTrxo1* transcripts were found ubiquitously in roots, rosette leaves, stems, and flowers of Arabidopsis (Fig. 2A) and its expression was practically constant during silique development (1 to >15 d after pollination, dap; Fig. 2B). To further study the expression of the *AtTrxo1* gene, stable transgenic lines of Arabidopsis (Col-0) with its promoter (–1047 bp) were produced that were transcriptionally fused to the reporter *uidA* gene, which encodes a β-glucuronidase (GUS) enzyme (*PAtTrxo1::uidA*). The length of the promoter used was selected taking into account the intergenic distance between the ATG translation initiation codon of *AtTrxo1* (*At2g35010*) and its preceding gene *At2g35000* in the Arabidopsis genome. GUS histochemical staining of these lines indicated that *AtTrxo1* was expressed in adult leaves preferentially in the vascular elements (Fig. 2Ca, Cb). In roots, GUS activity was detected at the initiation of the secondary roots and at the young internodes, as well as being expressed in the crown (Fig. 2Cc, Cd). GUS was also expressed at the floral primordia, the vascular bundle of the sepals and anthers, and at the stigma of flowers (Fig. 2Ce, Cf). In fruits, GUS was expressed at the base and at the upper part of the silique throughout all the stages analysed (Fig. 2Cg). Upon seed imbibition, GUS activity in the *PAtTrxo1::uidA* reporter lines was detected in the cotyledons, before and after radicle protrusion (Fig. 2Ch, Ci). Due to the high expression found in seeds and considering previous results showing pea Trx*o*1 as a part of the mitochondrial system responding to saline stress (Martí *et al.*, 2011), we focused on *AtTrxo1* expression during germination in both control and saline conditions as well as on its transcriptional regulation during this process. Firstly, we selected 100 mM NaCl as an appropriate treatment after an analysis of the germination kinetics at 10, 50, 75, 100, and 150 mM NaCl for the WT, but also of two KO *AtTrxo1* lines, as we describe below. The results for the two KO lines (KO1 and KO2) were found to be similar. A salt concentration of 100 mM NaCl was chosen because it showed the highest difference in germination rate between the WT and the KO lines whilst still allowing 100% germination (see Supplementary Fig. S2).

**Fig. 3.**
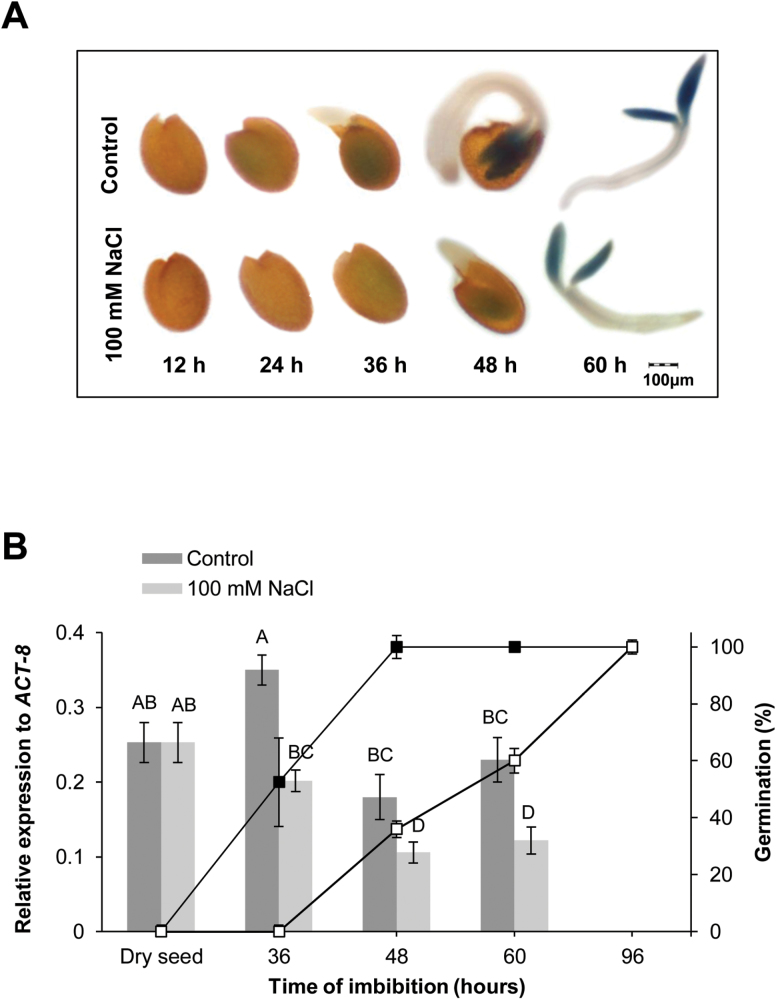
Expression of *AtTrxo1* during germination. (A) Histochemical localization of *PAtTrxo1::uidA* (–1047 bp) during germination in water (Control) and in the presence of salt (100 mM NaCl). (B) Expression analysis of the *AtTrxo1* gene by RT-qPCR upon seed germination in the absence (Control) or the presence of 100 mM NaCl. Data are means ±SE of three technical replicates of three biological samples. Different letters indicate that data are significantly different according to Tukey’s test (*P*<0.05). Percentage of germination is also indicated (Control, closed squares; 100 mM NaCl, open squares).

Analysis of GUS activity revealed an increase of expression of *AtTrxo1* in water-imbibed seeds during germination, mainly localized in cotyledons at 48 h, and a decrease upon imbibition in the presence of 100 mM NaCl (Fig. 3A). *AtTrxo1* expression was also analysed by RT-qPCR in imbibed seeds in 100 mM NaCl at 36, 48, and 60 h, using as controls seeds imbibed in water for the same period of time (Fig. 3B). *AtTrxo1* transcripts were especially abundant in dry seeds after 1 month of dry storage at 21 °C (after-ripened seeds), being one order of magnitude higher than in the other organs analysed (see Fig. 2A). In germinating control seeds, at 36 h of imbibition in water (*t*_50_ value when 50% of the seeds had germinated), the *AtTrxo1* transcript accumulation was similar to that found in dry seeds (Fig. 3B), decreasing thereafter until germination *sensu stricto* was completed (100% radicle protrusion at 48 h). In the presence of salt, a significant reduction of *AtTrxo1* transcript accumulation was found at each time-point analysed and a decrease was observed during germination, which was significantly delayed (Control *t*_100_=48 h; Salt *t*_100_=96 h).

### *Identification of evolutionary conserved* cis-*motifs in the* AtTrxo1 *gene promoter and of the TFs recognizing the conserved B2 domain*

**Fig. 4.**
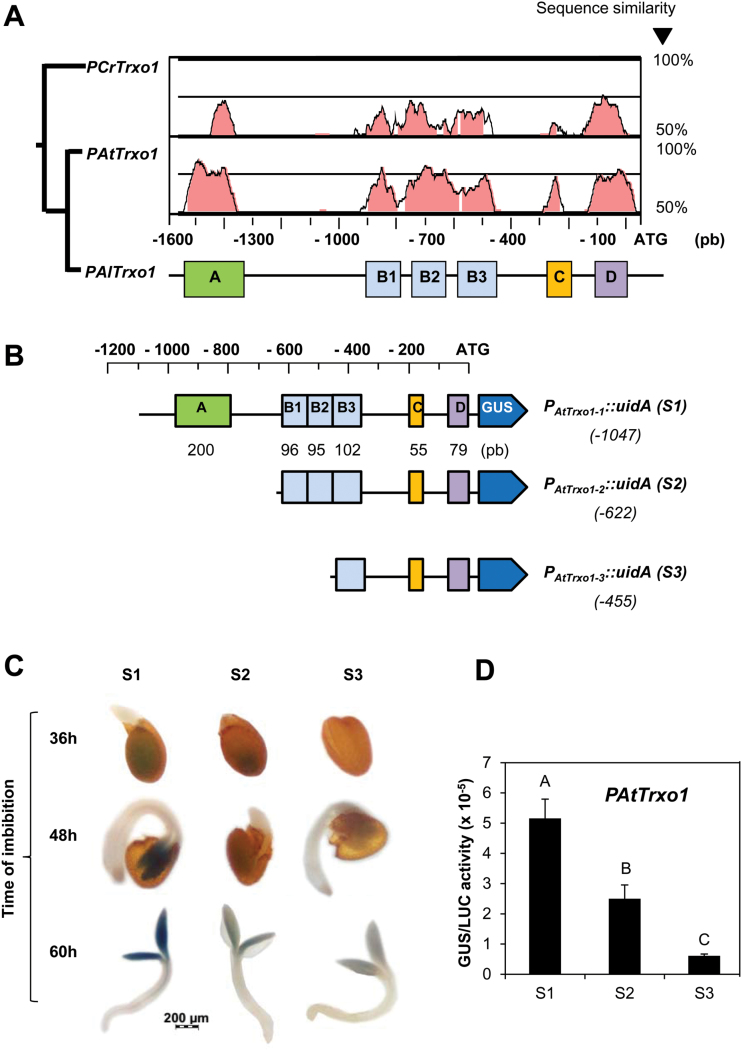
Influence of conserved domains A and B1+B2 of the *AtTrxo1* promoter on gene expression upon germination. (A) Identification of conserved *cis*-elements in orthologus *Trxo1* promoters in the Brassicaceae species *Arabidopsis thaliana* (At), *A. lyrata* (Al), and *Capsella rubella* (Cr). Shaded areas show conserved blocks. (B) Schematic representation of the three different promoter constructs of the *AtTrxo1* gene: S1 (–1047 bp), S2 (–622 bp), and S3 (–455 bp) fused to the reporter *uidA* gene (GUS) for use in transient expression assays. (C) Histochemical localization of GUS expression in transformed Arabidopsis plants with serial deletions of the *PAtTrxo1::uidA* construct (S1, S2, S3) during seed germination. (D) Quantification of GUS activity in tobacco leaves co-infiltrated with *Agrobacterium tumefaciens* containing the three different promoter constructs (S1, S2, S3) described in (B). Data are means ±SE of three independent experiments. Different letters indicate significant differences according to Tukey’s test (*P*<0.05).

The promoter regions of the *Trxo1* orthologous genes within the Brassicaceae species were searched *in silico* for conserved sequences (phylogenetic shadowing, Iglesias-Fernández *et al.*, 2013). The pair-wise alignment among three promoters analysed (*A. lyrata*, *C. rubella* and *A. thaliana*) showed several conserved blocks upstream of the ATG named motifs A, B, C, and D (see Fig. 4A, B).

In order to explore the functional relevance of these conserved motifs, a set of deletion constructs of the *AtTrxo1* promoter (*PAtTrxo1*) fused to the β-glucuronidase (GUS) reporter gene (*uidA*) were generated. These constructs are: *PAtTrxo1-1::uidA* (–1047 bp, S1) containing the whole promoter; *PAtTrxo1-2::uidA* (–622 bp, S2) lacking motif A; and *PAtTrxo1-3::uidA* (–455 bp, S3) deprived of motifs A, B1, and B2 (Fig. 4B). Transient expression assays by agro-infiltration of these constructs in *N. benthamiana* leaves showed a decrease in GUS expression with consecutive deletions (Fig. 4B, C). Homozygous transgenic Arabidopsis plants were generated with these constructs and GUS activity was evaluated during germination (Fig. 4D). The transgenic line with the *PAtTrxo1-1::uidA* construct showed that GUS activity was high in the cotyledons at 36 and 48 h of imbibition. Although the removal of the A motif decreased this activity, the S2 construct (*PAtTrxo1-2::uidA*) still retained 50% of the original GUS expression, and in the S3 construct (devoid of B1 and B2 elements) this expression was faint (Fig. 4B–D).

**Fig. 5.**
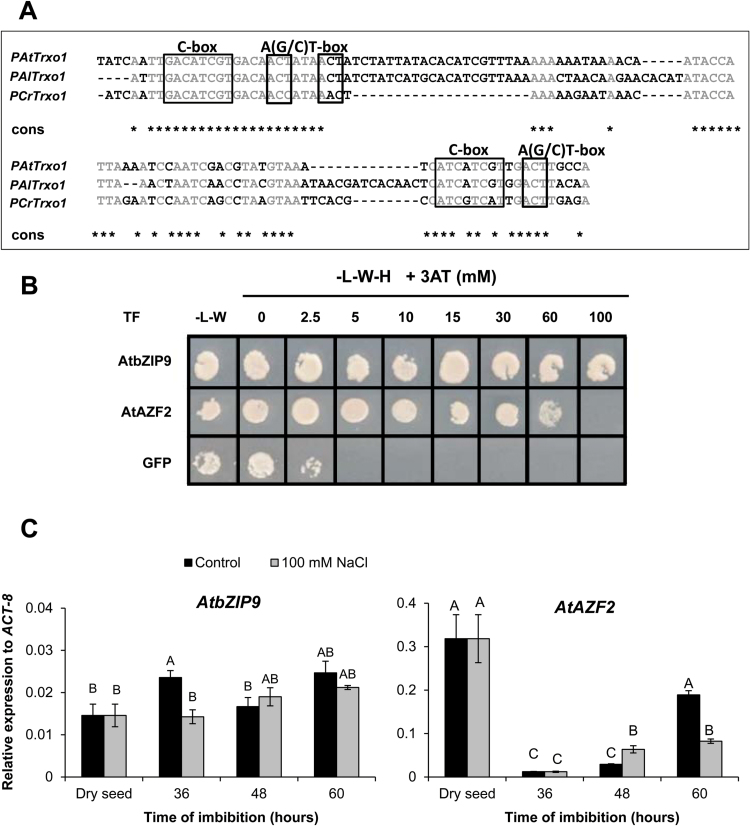
(A) Identification of the conserved *cis*-element, B2, in orthologous *AtTrxo1* promoters in the Brassicaceae species *Arabidopsis thaliana* (At), *A. lyrata* (Al), and *Capsella rubella* (Cr) by pair-wise alignment. The boxes indicate predictable *cis*-binding domains to AZF2 [A(G/C)T box] and bZIP9 (C-box). (B) AtAZF2 and AtbZIP9 bind to the *AtTrxo1*-B2-element in a yeast one-hybrid screening. Growth of diploid cells after mating of two yeast strains containing the *AtTrxo1-element–pTUY1H* construct and *pDEST22-AD:AtAZF2*, *pDEST22-AD:bZIP9*, or *pDEST22-AD:GFP* (negative control) on non-selective (–L–W) or selective medium (–L–W–H) with increasing concentrations of 3-AT. (C) Expression analysis of the *AtbZIP9* and *AtAZF2* genes (relative to *ACT-8*) as determined by RT-qPCR in dry seeds and upon germination in water or in 100 mM NaCl. Data are means ±SE of three technical replicates of three biological samples. Different letters indicate significant differences according to Tukey’s test (*P*<0.05).

The conserved *cis*-elements identified *in silico* as blocks B1 (96 bp) and B2 (95 bp; Figs 4B and 5A) were selected as baits for the screening of the arrayed library of Arabidopsis TFs in yeast (Castrillo *et al.*, 2011), and the AtbZIP9 (*At5g24800*) and AtAZF2 (*At3g19580*) that interacted with the B2 element were selected. Diploid yeast containing the plasmids *B2-element–pTUY1H* and *AtbZIP9-pDEST22* or *AtAZF2-pDEST22* were, respectively, able to grow in an auxothophic medium lacking histidine in the presence of up to 100 mM and 30 mM 3-AT a competitive inhibitor of the product of the *HIS3* gene (Fig. 5B). Moreover, in the web database PlantCare (http://bioinformatics.psb.ugent.be/webtools/plantcare/html/), in the block B2, a G-box (binding site for bZIP proteins) and an A(G/C)T box (binding site for AZF2 proteins) were predicted (Sakamoto *et al.*, 2004). To determine whether AtbZIP9 and AtAZF2 could be transcriptional regulators of the *AtTrxo1* gene during germination, the expression of the *AtbZIP9* and *AtAZF2* genes were analysed. Accumulation of *AtbZIP9* transcripts was high (Fig. 5C) at 36 h when 50% of the seeds had germinated (*t*_50_) and presented a similar pattern to *AtTrxo1* expression (Fig. 3B), in a manner compatible with AtbZIP9 being a transcriptional regulator of the *AtTrxo1* gene. In the presence of salt, expression of the *AtbZIP9* gene was lower than in control conditions only at 36 h (Fig. 5C) when no germinated seeds were observed (Fig. 3B). In contrast, the AZF2 transcript showed a significant increase in control conditions at 60 h but under salinity the increase was significant at 48 h (Fig. 5C), when 40% of the seeds were germinated (Fig. 3B), which is compatible with a role as a negative regulator of the *AtTrxo1* gene.

**Fig. 6.**
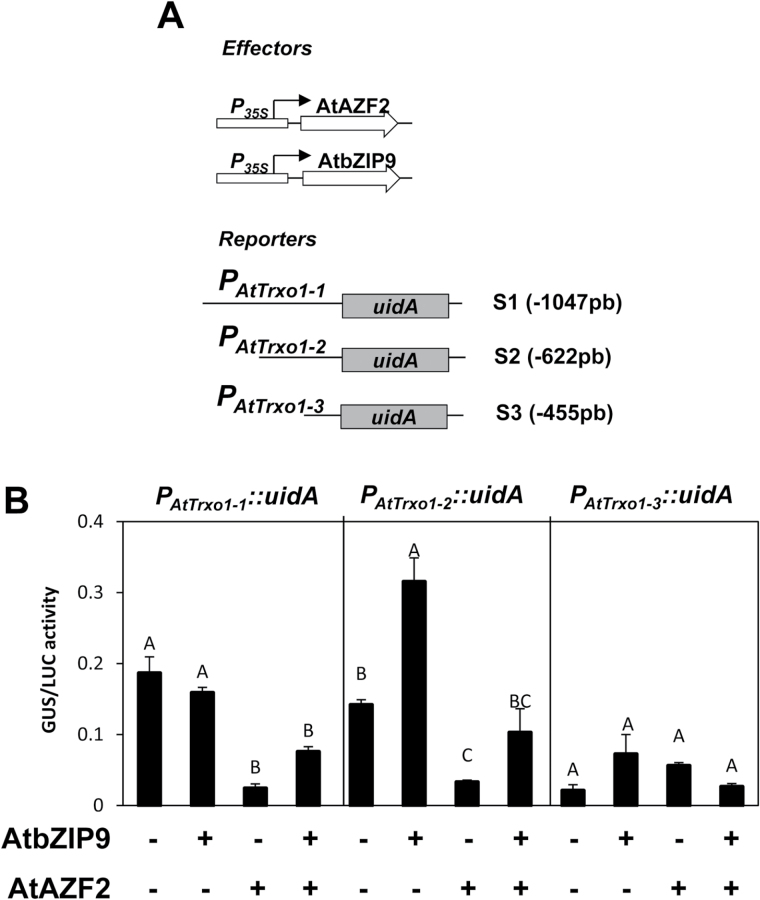
Trans-activation assays using as effectors the TFs AtbZIP9 and AtAZF2, and as reporters the *AtTrxo1* gene promoter driven by the expression of the *uidA* gene (GUS activity). (A) Schematic representation of the effector and reporter contructs used in the analysis. (B) Agro-infiltration of the effector and the reporter combinations indicated in tobacco leaves. The relative amounts of reporter and effector plasmids used in these assays correspond to the 1:1 ratio. GUS activity was relative to luciferase (LUC) activity, which was used as internal control. Data are means ±SE of three biological replicates. Different letters indicate significant differences according to Tukey’s test (*P*<0.05).

The interaction between AtbZIP9 and AtAZF2 with the B2 element was further investigated *in planta* (Fig. 6) using a tobacco transient expression system (*N. benthamiana* leaves). As reporter constructs, the promoter fragments S1, S2, and S3 fused to the *uidA* (GUS) gene were used (*PAtTrxo1-1::uidA*, *PAtTrxo1-2::uidA*, and *PAtTrxo1-3::uidA*, Figs 4B and 6A); as effectors, plasmid constructs were used where the AtbZIP9 and /or the AtAZF2 coding sequences were under the control of the 35S CaMV promoter (*P35S::AtbZIP9*, *P35S::AtAZF2*) (Fig. 6A). A construct containing the LUC-encoding ORF (*P35S::LUC*) was used as an internal control for the transformation experiments. Co-transformation of *P35S::AtbZIP9* and reporters enhanced the GUS activity driven by the S2 construct, but not by the S1 construct (Fig. 6B). Although the two constructs contain the B2 motif, only the S1 contains the A element. Moreover, GUS activity was not enhanced in the S3 construct, which is devoid of the A, B1, and B2 motifs. Co-transformation of *P35S::AtAZF2* and the S1 and/or S2 constructs, both containing the B2 element, diminished the GUS activity driven by the S1 and S2 promoters; when both effectors were transfected, the repressor effect of AtAZF2 counteracted the transcriptional activator effect of AtbZIP9 (Fig. 6B).

### Growth and germination of Arabidopsis lacking Trxo1 in saline conditions

**Fig. 7.**
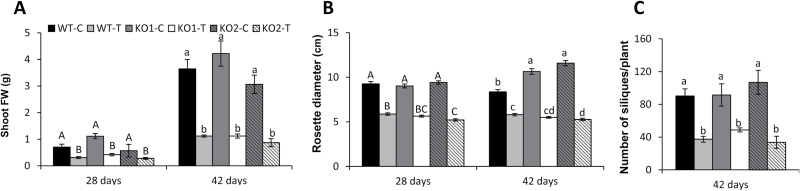
Physiological characterization of the wild-type (WT) and two knock-out (KO) *AtTrxo1* mutants after 28 and 42 d growing in the absence (control, C) or presence (treated, T) of 100 mM NaCl. The shoot fresh weight (A), rosette diameter (B), and number of siliques per plant (C) were determined in plants grown in a growth chamber as described in the Material and Methods. Data are means ±SE of at least three independent experiments. Different letters (capital letters at 28 d and lower-case letters at 42 d) indicate that data at each time point are significantly different according to Tukey’s test (*P*<0.05).

In order to gain further information on the functions of Trx*o*1 and its potential role during plant development and seed germination, we analysed the growth of wild-type Arabidopsis plants and two T-DNA insertion mutant lines in the *thioredoxin-o1* gene (*At2g35010*) from the Salk collection (KO1, SALK_143294C; KO2, SALK_042792). The insertion in the *Trxo1* gene was mapped to the first intron and the lack of *AtTrxo1* expression in germinating seeds of these mutants (at 36 h) was confirmed by RT-qPCR analysis (Supplementary Fig. S3A, B). We then studied the growth of plants under saline conditions and we also examined germination in the presence of NaCl. After 28 and 42 d of growth in control conditions, wild-type and both KO mutant plants showed similar fresh weights (Fig. 7A), and they also showed similar rosette diameter at 28 d, although this parameter was significantly higher in the two KO lines at 42 d of growth (Fig. 7B). At 42 d of growth, the number of siliques per plant was not significantly different between the mutants and the WT plants (Fig. 7C). Analysing the response of plants grown under salt conditions, we found no apparent differences between WT and KO mutant plants, although a strong effect at 42 d was observed in all the plants with loss of chlorophyll and senescence symptoms (Supplementary Fig. S4). Treatment with 100 mM NaCl caused a similar decrease in all the physiological parameters measured in the three genotypes (Fig. 7). With this treatment, no differences were found in the fresh weight between WT and KO mutants at 28 and 42 d of development and the number of siliques was similar, while the rosette diameter was found to be smaller in the KO2 plants.

**Fig. 8.**
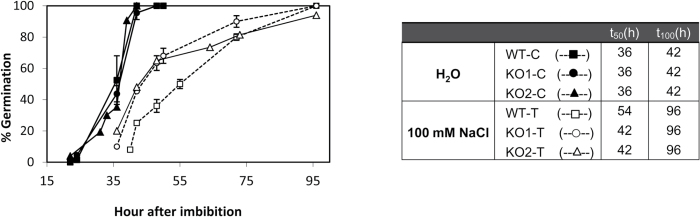
Germination time course of seeds imbibed in water (control C, closed symbols) or in 100 mM NaCl (treated T, open symbols) in the wild-type (WT, squares), KO1 mutant (circles), and KO2 mutant (triangles). The *t*_50_ value (time required for radicle emergence in 50% of seeds) is indicated in the table. Data are means ±SE of three technical replicates of six biological samples.

Analysing germination in control conditions, and as previously described for the KO2 mutant by Daloso *et al.* (2015), we found that seeds of both KO *AtTrxo1* lines failed to show any differences in germination rate compared to WT water-imbibed seeds (100% of germination at 42 h; *t*_50_=36 h; Fig. 8). When 100 mM NaCl was used as the imbibition medium, the germination in the KO *AtTrxo1* mutants was faster (*t*_50_=42 h) than that of the WT seeds (*t*_50_=54 h), although all of them reached 100% germination at 96 h (Fig. 8).

### *Oxidative parameters and* Prx *and* Srx *gene expression during germination in saline conditions*

**Fig. 9.**
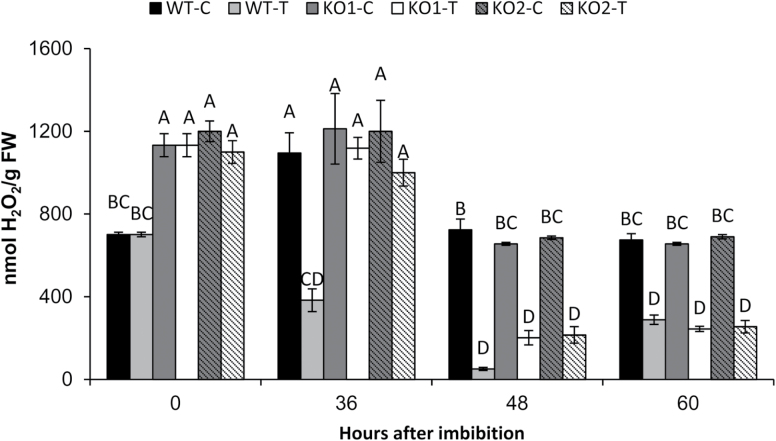
Hydrogen peroxide content (g^–1^ FW) in dry seeds (0 h) and germinating seeds of the wild-type (WT) and KO *AtTrxo1* (KO1 and KO2) in control (C) water conditions or in the presence of 100 mM NaCl (treated, T). Data are means ±SE of three technical replicates of three biological samples. Different letters indicate significant differences according to Tukey’s test (*P*<0.05).

Since H_2_O_2_ plays a central role in redox homeostasis, we analysed the H_2_O_2_ content in seeds at different times of germination in WT and KO *AtTrxo1* lines (KO1 and KO2), in water and in 100 mM NaCl. As shown in Fig. 9, dry seeds of the KO lines had a 1.5-fold higher content than the WT seeds, while upon germination in water (36, 48, 60 h) no significant differences were observed. In water, a high H_2_O_2_ content was observed at 36 h, it decreased at 48 h and was maintained at 60 h in all the genotypes. In saline conditions, the H_2_O_2_ content at 36 h in the WT plants decreased to approximately half of that found in control conditions, while it remained at the same value as the dry seeds in the KO mutants, in which the content represented a three-fold increase as compared with the WT seeds under these stress conditions. At 48 h, the H_2_O_2_ diminished drastically in both the WT and KO mutants, and this content remained without significant changes in both types of seeds at 60 h.

Since the patterns of germination rate and H_2_O_2_ content were similar in both KO1 and KO2 seeds, we chose the KO1 mutant to measure other oxidative parameters. Measurement of protein oxidation (carbonyl protein content) and lipid peroxidation (thiobarbituric acid reactive substances content, TBARS), as markers of oxidative stress, in the seeds of both lines under control and saline conditions revealed no significant changes between WT and mutant seeds over time under both conditions, although the presence of 100 mM NaCl decreased the lipid peroxidation level in dry seeds of both lines (Supplementary Fig. S5).

Since the Trx*o*1/PrxIIF/Srx system has been described as important for ROS homeostasis in mitochondria (Iglesias-Baena *et al.*, 2011), the expression of *AtPrxIIF* and *AtSrx* genes during germination was examined both in water and in 100 mM NaCl. In dry seeds, the *AtPrxIIF* and *AtSrx* transcript content was higher in the WT than in the KO1 *AtTrxo1* mutant, and both genes decreased their expression upon imbibition, but no significant differences between the lines were observed in control or even in saline conditions (Supplementary Fig. S6).

## Discussion

The Arabidopsis genome has several thioredoxin genes. Laloi *et al.* (2001) first identified an *AtTrxo1* gene encoding a thioredoxin located in mitochondria. Martí *et al.* (2009, 2011) found in pea leaves a double location for the *PsTrxo1* encoding protein in mitochondria and in the nucleus, and demonstrated its participation in saline stress. The gene *AtTrxo1* is expressed ubiquitously in the vascular elements of leaves and roots, and this expression is particularly important in dry and germinating seeds, with a high transcript accumulation at 36 h of imbibition, as was shown by RT-qPCR and GUS expression assays. This localization in the seed is similar to that of other thioredoxins, such as Trx*f* and Trx*m* from pea seedlings and Trx*h* from barley seeds (Shahpiri *et al.*, 2008; Fernández-Trijueque *et al.*, 2012). The implication of these cytoplasmic and chloroplastic Trxs in germination has been described previously (Montrichard *et al.*, 2009; Pulido *et al.*, 2009), but there is no information concerning the participation of mitochondrial AtTrx*o*1 in germination. The reactivation of metabolism that occurs during seed germination generates an important quantity of ROS and produces an increase in the content of diverse antioxidant compounds, such as flavonoids, phenols, ascorbate (ASC), and reduced glutathione (GSH), as well as increases in the expression of *Trx*, *Prx*, and *CAT* genes (Simontacchi *et al.*, 1993; De Gara *et al.*, 1997; Yang *et al.*, 2001; De Tullio and Arrigoni, 2003). Our data indicate a high *AtTrxo1* expression in cotyledons, similar to that reported for other Trxs, such as Trx*h*6 in *Medicago truncatula* (Renard *et al.*, 2011), with a role related to the regulation of specific targets. In addition, Trx*h*1 and, to a lesser extent, Trx*h*2 are abundant in both embryonic axes and cotyledons, so the different spatial distribution of the isoforms of Trx*h* in *Medicago* suggests that they play different roles during germination, which might be related to the maintenance of the redox homeostasis during this process. In previous work, both Trx*h*3 and *h*4 were found in dry seeds of pea only in embryo axes (*h*4) or in both axes and cotyledons (*h*3), indicating that they were synthesized before germination. In contrast, they showed similar expression profiles upon imbibition, with a strong induction of expression in axes after radicle protrusion (46 h) and in cotyledons just before and after radicle protrusion (22 and 46 h; Montrichard *et al.*, 2003). The authors suggested different roles for these Trxs during germination, such as reserve mobilization or protection against ROS. In fact, several proteases, α-amylases, and their inhibitors in the endosperm and embryo are mainly oxidized in dry seeds and are reduced to the sulfydril state after imbibition, increasing their solubilization and susceptibility to proteolysis (Lozano *et al.*, 1996; De Gara *et al.*, 2003; Serrato and Cejudo, 2003). In addition, in the dicotyledonous *Medicago truncatula*, and in the monocotyledonous *Triticum* sp. *and Hordeum vulgare*, the majority of the proteins susceptible to redox modification at the beginning of germination are targets of Trx (Wong *et al.*, 2004; Alkhalfioui *et al.*, 2007; Montrichard *et al.*, 2009).

Although available information about transcriptional regulation of plant thioredoxin genes is scarce, CCA1 and DOF7 TFs have been identified as regulators of *Pisum sativum* chloroplast *Trxf* and *Trxm1*, encoding genes that respond to the circadian cycle and to glucose levels (Blazquez *et al.*, 2011; Barajas-López *et al.*, 2012). In addition, the WRKY6 TF is a positive regulator of cytosolic At*Trxh*5 expression, which is also mediated by ROS under oxidative stress conditions. Moreover, *AtTRXh5* was up-regulated in plants overexpressing *WRKY6*. This regulation is specific to the thioredoxin *h* family (Laloi *et al.*, 2004). However, no information is available regarding the transcriptional regulation of *Trxo1*. To rectify this, the *cis–trans* transcriptional regulatory code of *AtTrxo1* was established by phylogenomic analyses of orthologous *Trxo1* gene promoters within the Brassicaceae family coupled with screenings of an arrayed library of Arabidopsis TFs (Castrillo *et al.*, 2011). By this method, the conserved B2-element, the AtbZIP9 and the AtAZF2 TFs, were selected.

AtbZIP9 belongs to the basic leucine zipper C-subfamily of TF proteins that includes AtbZIP10, AtbZIP25, and AtbZIP63 (Jakoby *et al.*, 2002; Lara *et al.*, 2003). The importance of the conserved motifs in the *AtTrxo1* promoter has been demonstrated *in planta* by transient expression in agro-infiltrated *N. benthamiana* leaves and by stable GUS expression (*uidA* gene) driven by sequential deletions of *AtTrxo1* promoter constructs. These experiments indicated the relevance of block B2 as an interactor with the AtbZIP9 TF, a transcriptional activator of the *AtTrxo1* gene. The co-expression of the *P35S::AtbZIP9* and the *PAtTrxo1-2::uidA* (containing the B2 element) constructs in *N. benthamiana* leaves revealed the activator effect of AtbZIP9 over the *AtTrxo1* promoter, and this effect disappeared when the B2 motif was deleted. Moreover, when the A motif was present (*PAtTrxo1-1::uidA*; Fig. 6B), the activation effect of AtbZIP9 was not observed, perhaps indicating an accession difficulty of this TF to its interacting B2 *cis*-motif. In addition, during germination the *AtbZIP9* gene displayed a pattern of expression compatible with being a transcriptional activator of the *AtTrxo1* gene. Other TF genes from the bZIP C-subfamily, namely *AtbZIP10* and *AtbZIP25*, have been found to have expression patterns during seed maturation that temporally and spatially match with those of the seed storage protein genes (Lara *et al.*, 2003). Nevertheless, no effect in the *AtbZIP9* expression profile was observed in germination under salinity, although the decrease of *Trxo1* expression with time of seed imbibition indicated that under salinity another TF with transcriptional repressor activity must be involved.

AtAZF2 belongs to the Cys-2/His-2-type zinc finger proteins that they are induced in plants by dehydration, salinity, cold stress, and ABA treatment (Sakamoto *et al.*, 2004). Transient assays *in planta* with co-expression of the *P35S::AZF2* and *PAtTrxo1-1::uidA* and *PAtTrxo1-2::uidA* constructs revealed the repressor effect of AtAZF2 over the *AtTrxo1* gene. In addition, expression analysis of *AtAZF2* during germination confirms the behaviour of this gene as a repressor of *AtTrxo1*, mainly under salinity. This behaviour has been already reported in seeds, where AZF2 acts as a repressor of several genes under salt stress (Sakamoto *et al.*, 2004; Drechsel *et al.*, 2010; Kodaira *et al.*, 2011).

**Fig. 10.**
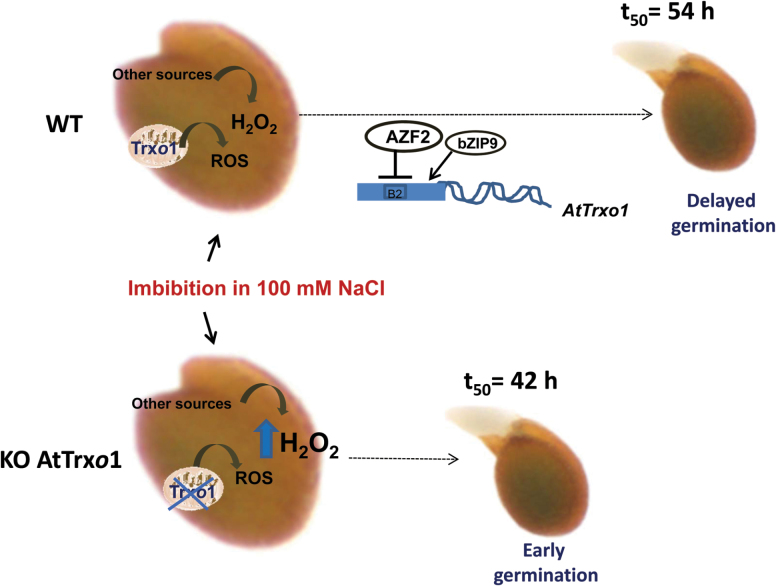
Proposed model of the germination of Arabidopsis wild-type (WT) and KO AtTrx*o*1 mutant seeds in the presence of 100 mM NaCl. The production of H_2_O_2_ by the mitochondria and other sources in the presence of NaCl accelerates germination of imbibed seeds and is greater (2–3 times) in KO Trx*o*1 mutants than in the WT seeds. *t*_50_, time when 50% of the seeds have germinated. AZF2 is a transcriptional repressor (line ending in bar) and bZIP9 is an activator (arrow).

Trx*o*1 mutants have been used to corroborate a role for the Trx system in regulating different metabolic processes in mitochondria, although no extreme phenotype has been described, possibly due to the redundancy or overlapping functions with mitochondrial or cytosolic proteins as glutaredoxins (Daloso *et al.*, 2015). Among abiotic stresses, salinity is one of the most important unfavorable conditions for plant yield and growth, and redox systems are considered as key players for stress sensing and signal transduction pathways (Lázaro *et al.*, 2013). In our study on the behavior of KO *AtTrxo1* plants growing in the presence of 100 mM NaCl, the lack of Trx*o*1 seems to be compensated for, as evidenced by the plant growth of Trx*o*1 insertion lines not being significantly affected under this stress situation compared to the WT plants, although this behaviour was more evident in the KO1 than in the KO2 plants, as shown by the rosette diameter. Production of ROS, particularly H_2_O_2_, increases during seed maturation in sunflower seeds, and the subsequent ability to germinate depends on a critical accumulation of this compound (Bailly *et al.*, 2008). An increase in H_2_O_2_ has been also reported in other species under salinity conditions and it has been described as an inducer of earlier germination (Puntarulo *et al.*, 1991; Hernandez *et al.*, 2001; Lin *et al.*, 2013). In our analyses, the H_2_O_2_ levels were higher in dry seeds of the KO *AtTrxo1* than in the WT, although there was no detectable difference either in the germination kinetics in water or in protein oxidation or lipid peroxidation between the WT and the KO mutant. Additionally, in both of these seeds, the expression of *AtPrxIIF* and *AtSrx* did not differ significantly during germination in either water or NaCl. This behaviour was different to that found in pea plants, where *Trxo1* and *PrxIIF* expression is increased in response to short-term salt stress (Barranco-Medina *et al.*, 2008; Martí *et al.*, 2011), pointing to the heterogeneity of response of the antioxidant system depending on the salt sensitivity of the cultivars, the NaCl concentration, and the duration of the stress (Lázaro *et al.*, 2013). Salinity in imbibed seeds usually produces a delay in germination, as occurred in our experiments, although we found that KO *AtTrxo1* seeds had higher H_2_O_2_ content at the beginning of germination and a faster germination rate than those of the WT (see *t*_50_ values in Fig. 8). Figure 10 is presented as a summary illustration of our main results in saline conditions, including the participation of AtbZIP9 and AtAZF2 TFs in the regulation of the *AtTrxo1* gene. Among other ROS, H_2_O_2_ is known to accumulate during imbibition and early stages of germination, with mitochondria being essential producers (Zhang *et al.*, 2014). The differential H_2_O_2_ peak in the mutant line may be one factor in the early germination shown under salt stress, and the lack of lipid or protein oxidation could be related with the different roles of H_2_O_2_ in cell wall growth and cross-talk with NO and hormones such as ABA and GA (Wojtyla *et al.*, 2016). Similar changes as regards bringing forward or delaying germination have been described for other redox proteins, including Trxs. The fact that this divergence in the germination pattern between WT and KO seeds basically occurs in the face of saline stress may reflect a specific role for Trx*o*1 in the germination of seeds exposed to salt stress, which may in turn be related to specific Trx*o*1 targets. This behaviour is similar to some antioxidant enzymes, such as symplastic ascorbate oxidase from tobacco and Arabidopsis (Yamamoto *et al.*, 2005), and the behaviour found in Arabidopsis RNA interference lines of the barley ortholog of *AtPER1* (encoding a 1 Cys-Prx), which germinated earlier than WT seeds under salinity, whereas the over-expression of *AtPER1* caused germination to be delayed (Haslekås, 2003).

In summary, these results indicate a role for the *AtTrxo1* gene in seed germination that is more evident in 100 mM NaCl, where Trx*o*1 could act as a possible sensor of saline stress and an inducer of H_2_O_2_ accumulation, independently of other ROS parameters (*PrxIIF* and *AtSrx* gene expression, protein oxidation, or lipid peroxidation). In addition, for the first time, the transcriptional regulation of this gene has been investigated. Two transcription factors have been identified, bZIP9 and AZF2, that show a positive and negative role, respectively, over *Trxo1* expression during germination. Further studies that are focused on determining the specific Trx*o*1 target proteins and/or its involvement in redox signaling pathways will help to establish the mechanism by which Trx*o*1 is acting during germination.

## Supplementary Material

Supplementary Data
