# Supplementary material for: Mitochondrial AtTrxo1 is transcriptionally regulated by AtbZIP9 and AtAZF2 and affects seed germination under saline conditions
Source: J Exp Bot. 2017 Feb 10;68(5):1025–38. doi: 10.1093/jxb/erx012 (PMC5441863; doi:10.1093/jxb/erx012)
Supplement: Supplementary Data [file erx012_Supplementary_Data.zip › supplementary_figures_S1_S6_table_S1.pdf]

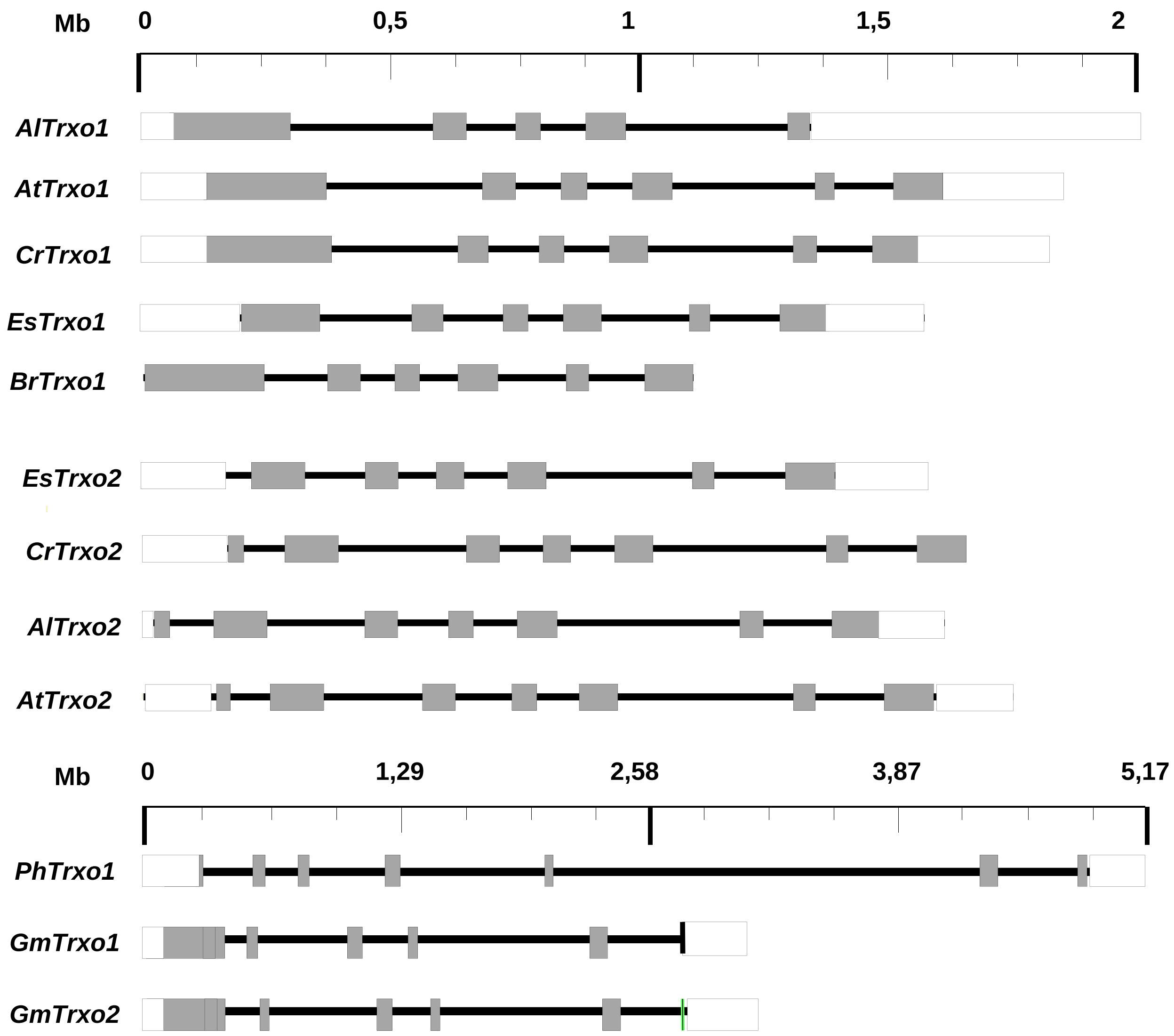

Fig. S1. Predicted intron-exon gene structures of *AtTrxo1* and *AtTrxo2* and orthologous genes presented in Fig 1A. Introns are represented with lines and exons with rectangles.

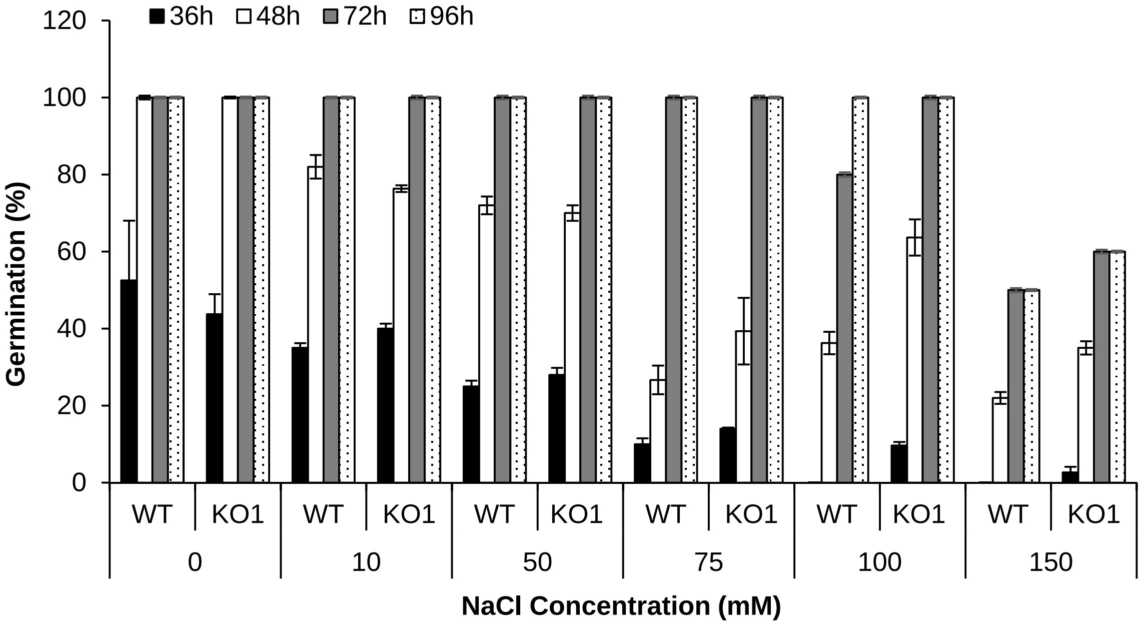

Fig. S2. Germination percentage of wild-type (WT) and KO *AtTrxo1* (KO1) in control water conditions and in the presence of different NaCl concentrations. Data are means ± standard error of three technical replicates of three biological samples.

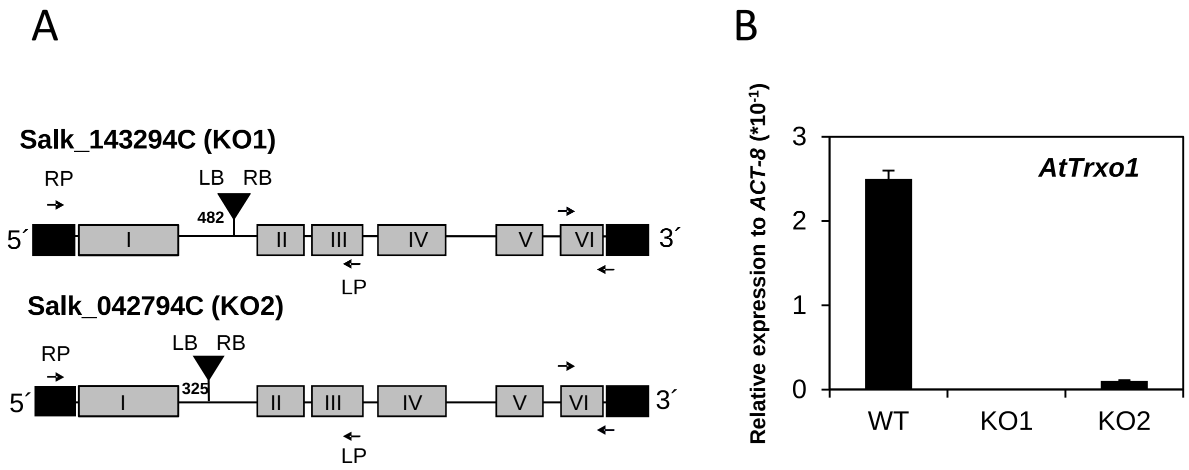

Fig. S3. (A) Position of the T-DNA insertion in the first intron: Intron (lines)-exon (rectangles) distribution. Inverted triangle represents the location of the T-DNA with the left (LB) and right (RB) borders. Black arrows represent the primers for the qPCR assay, **(B)** Expression of *AtTrxo1* gene in 36 h-imbibed seeds in the wild-type and KO1 and KO2 mutants.

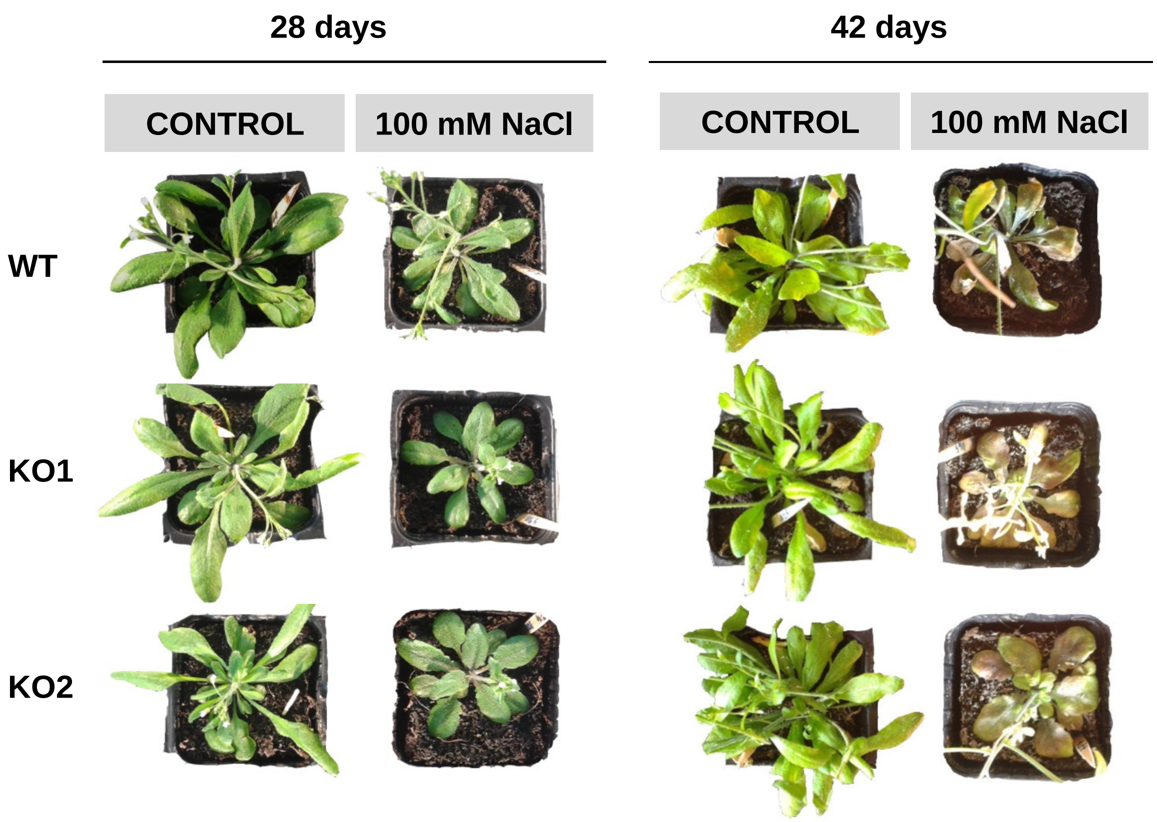

Fig. S4. Representative wild-type and two KO *AtTrxo1*mutant plants after 28 and 42 days growing in the absence (control) or presence of 100 mM NaCl.

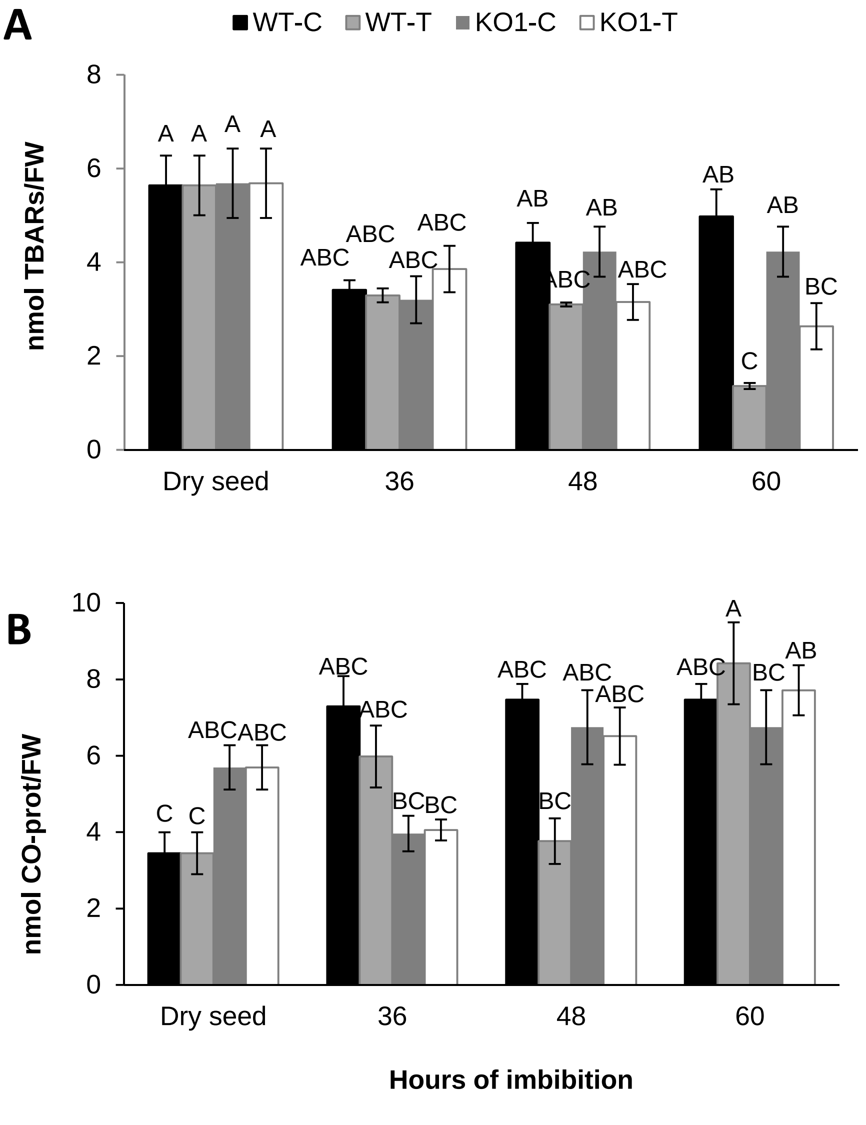

Fig. S5. (A) Lipid peroxidation (thiobarbituric acid reactive substances, TBARS) and **(B)** protein carbonyl oxidation (CO-protein) in dry seeds and during germination of wild-type (WT) and KO *AtTrxo1* (KO1) in the absence (control, C) or presence (treated, T) of 100 mM NaCl. Data are means ± standard error of three technical replicates of three biological samples. Different letters indicate that data are significantly different by Tukey's test at P<0.05. FW: fresh weight.

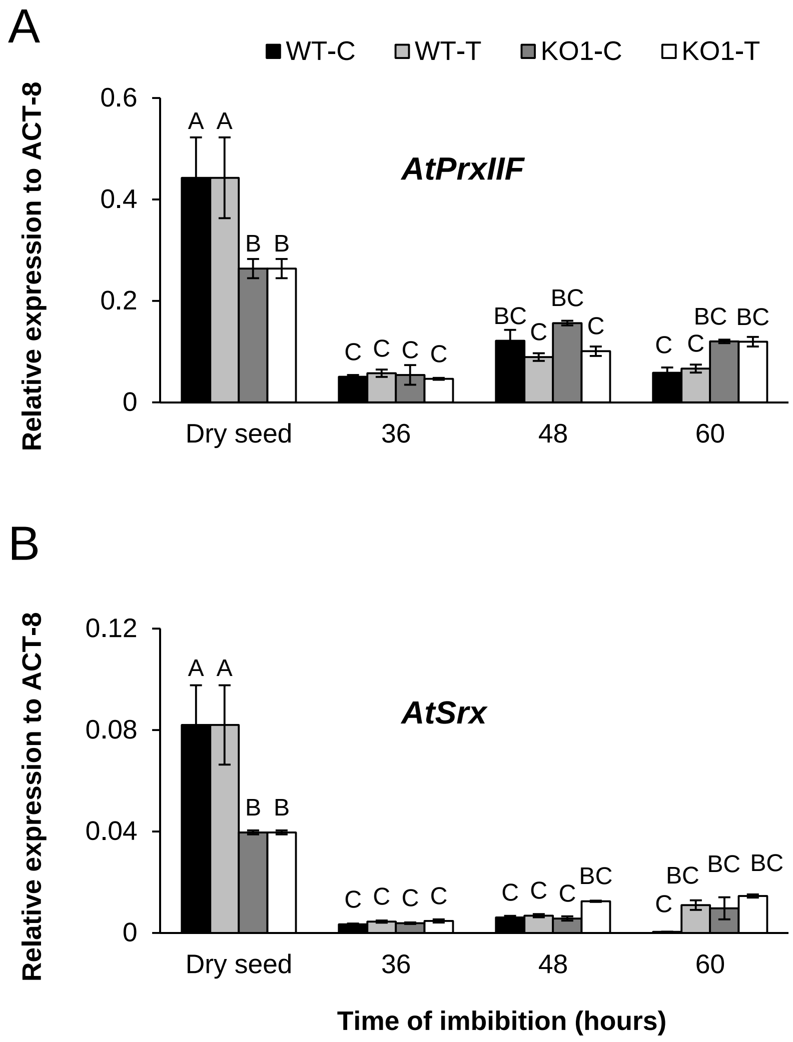

Fig. S6. Expression of **(A)** peroxiredoxin *AtPrxIIF* and **(B)** sulfiredoxin *AtSrx* genes by RT-qPCR in dry seeds and during germination of wild-type (WT) and KO *AtTrxo1* (KO1) in the absence (control, C) or presence (treated, T) of 100 mM NaCl. Data are means ± standard error of three technical replicates of three biological samples. Different letters indicate that data are significantly different by Tukey's test at $P<0.05$.

Table S1. Sequences of primers used in the RTqPCR analysis, cloning, and genotyping of T-DNA insertion mutant lines.

**Name**	**Locus**	**Sequences 5´-3´**	**Primer efficiency (%)**	**Amplicon dissociation t[a]**
**RTqPCR primers**				
S-AtTrxo1	*At2g35010*	TCGAAGAAAGGGGAGGTTG	89.37	76.3ºC
AS-AtTrxo1	*At2g35010*	CACTTGTAGAGCTGTTCCATGAG		
S-ACT8-RNA	*At1g49240*	GGTCGTACAACCGGTATTGT	Graeber *et al*. (2011)	77.8ºC
AS-ACT8-RNA	*At1g49240*	GAAGAGCATACCCCTCGTA		
S-AtAZF2	*At3g19580*	CCTGAACTCAGCCTTCATCA	103	77.5ºC
AS-AtAZF2	*At3g19581*	TGACTTGGTCGTGATCGGTC		
S-AtbZIP9	*At5g24800*	CATCTGGCCATGACTCTCTTT	104	78.0ºC
AS-AtbZIP9	*At5g24801*	AACGAACATGCTGCTTTCAT		

**Name**	**Locus**	**Sequences 5´-3´**
**Cloning primers**		
AS-pAtTrxo1-S1-attB2	*At2g35010*	CAAGAAAGCTGGGTCCTTCATCACTCGAGCTTTTAG
S-S2-uidA/pAtTrxo1	*At2g35010*	AAAAAAGCAGGCTTCGTATTATCTCTAACCATT
S-S3-uidA/pAtTrxo1	*At2g35010*	AAAAAAGCAGGCTTCCCAATCGACGTATGTAAATC
S-S4-uidA/pAtTrxo1	*At2g35010*	AAAAAAGCAGGCTTCCACAAGATCCAATCATTAC
AS-uidA/pAtTrxo1	*At2g35010*	CAAGAAAGCTGGGTCCTTCATCACTCGAGCTTTTAG
attB1	*GATEWAY*	GGGGACAAGTTTGTACAAAAAAGCAGGCTTC
attB2	*GATEWAY*	GGGGACCACTTTGTACAAGAAAGCTGGGTC
S-pAtTrxo1-B2-XmaI	*At2g35010*	AGCCCGGGTATCAATTGACATCGTGACA
AS-pAtTrxo1-B2-XbaI	*At2g35010*	TCTCTAGATTTACATACGTCGATTGGAT
**Genotyping primers**		
AS/ SALK_143294	*At2g35010*	AATCATCATCGTTGACTTGCC
S/ SALK_143294	*At2g35010*	ACACATCCACTTAGCGTGAGG
AS/SALK_04042792	*At2g35010*	TCGAGTGATGAAGGGAAATTG
S/SALK_04042792	*At2g35010*	AAATCCCGCCCTACAGATATG
LBb1.3	*Left border primer*	ATTTTGCCGATTTCGGAAC
